# Leaflet‐Specific Structure and Dynamics of Solid and Polymer Supported Lipid Bilayers

**DOI:** 10.1002/anie.202423784

**Published:** 2025-04-07

**Authors:** Narain Karedla, Falk Schneider, Jörg Enderlein, Tao Chen

**Affiliations:** ^1^ Third Institute of Physics – Biophysics Georg August University Friedrich‐Hund‐Platz 1 Göttingen 37077 Germany; ^2^ The Rosalind Franklin Institute Harwell Campus Didcot OX11 0FA UK; ^3^ Kennedy Institute of Rheumatology University of Oxford Roosevelt Drive Oxford OX3 7LF UK; ^4^ Translational Imaging Center University of Southern California Los Angeles CA 90089 USA; ^5^ Biomedical Sciences Warwick Medical School University of Warwick Coventry CV4 7AL UK; ^6^ Cluster of Excellence “Multiscale Bioimaging: from Molecular Machines to Networks of Excitable Cells” (MBExC) Universitätsmedizin Göttingen Robert‐Koch‐Str. 40 Göttingen 37075 Germany

**Keywords:** Fluorescence correlation spectroscopy, Fluorescence lifetime microscopy, Graphene‐induced energy transfer, Supported lipid bilayer, Tethered PEGylated SLB

## Abstract

Polymer‐supported or tethered lipid bilayers serve as versatile platforms for mimicking plasma membrane structure and dynamics, yet the impact of polymer supports on lipid bilayers remains largely unresolved. In this study, we introduce a novel methodology that combines graphene‐induced energy transfer (GIET) with line‐scan fluorescence lifetime correlation spectroscopy (lsFLCS) to examine the structural and dynamic properties of lipid bilayers. Our findings reveal that polymer supports markedly influence both the structural parameters, such as the membrane height from the substrate, its thickness, as well as dynamic properties, including leaflet‐specific diffusion coefficients and interleaflet coupling. These findings highlight the complex interplay between a polymer support and the lipid bilayers. By resolving leaflet‐specific diffusion and heights of the two leaflets from the substrate, this study emphasizes the potential of GIET‐lsFLCS for probing membrane dynamics and structure. These insights significantly advance the understanding and application of polymer‐supported membranes across diverse research contexts.

## Graphical abstract

The manuscript investigates the structural and dynamic properties of polyethylene glycol‐supported lipid bilayers using a method based on graphene‐induced energy transfer and line‐scan fluorescence lifetime correlation spectroscopy, providing subnanometer spatial resolution and millisecond temporal resolution within a single experiment.

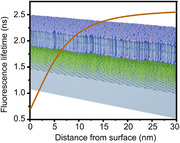

## Introduction

Plasma membranes host a myriad of essential proteins and lipids crucial for cellular functions. However, the intricate composition and inherently active, nonequilibrium nature of native plasma membranes—driven by energy‐dependent processes such as lipid recruitment, active transport, and cytoskeletal remodeling—pose significant challenges for isolating and studying individual components. Supported lipid bilayers (SLBs) provide a valuable bottom‐up alternative, enabling the preparation of simplified membrane models that can be investigated using a wide range of surface‐sensitive techniques.^[^ ^1^ ^]^ These techniques include neutron reflectometry (NR),^[^ ^2^, ^3^ ^]^ atomic force microscopy (AFM),^[^ ^4^, ^5^, ^6^ ^]^ surface plasmon resonance spectroscopy (SPR),^[^ ^4^, ^7^, ^8^ ^]^ electrochemical impedance spectroscopy (EIS),^[^ ^9^, ^10^, ^11^ ^]^ and fluorescence microscopy.^[^ ^12^, ^13^, ^14^, ^15^, ^16^, ^17^, ^18^ ^]^ Owing to their versatility, SLBs have become well‐established as model biomimetic systems in a wide range of biophysical and biological studies. They are invaluable tools for exploring the self‐assembly and dynamics of lipids and proteins, providing crucial insights into these complex biological processes.

SLBs are composed of two closely coupled leaflets, the proximal leaflet, which faces the solid support, and the distal leaflet on the opposite side. The proximal leaflet is separated from the substrate surface by a thin hydration layer, 1–2 nm in thickness approximately, that acts as a lubricant, enabling the lipids to maintain their fluidity.^[^ ^19^ ^]^ Recent experimental findings, however, suggest that a small fraction of labeled lipids in the SLB transiently adsorbed onto the substrate.^[^ ^20^ ^]^ Consequently, the dynamics of labeled lipids can only be described accurately by accounting for this partial adsorption.^[^ ^1^, ^20^, ^21^ ^]^ In contrast, the distal leaflet is influenced by interleaflet coupling with the proximal leaflet, a key factor that shapes the overall structure and dynamics of the bilayer.^[^ ^15^, ^22^, ^23^, ^24^ ^]^ The close proximity of the solid support poses additional challenges when studying membrane‐embedded proteins incorporated into SLBs. Undesired interactions with the solid support can impair protein function and, in some cases, lead to protein denaturation.^[^ ^1^, ^25^, ^26^ ^]^ To overcome these challenges, many studies have introduced a soft polymer “cushion” between the membrane and the solid support, forming polymer‐supported lipid bilayers (p‐SLBs). This polymer layer increases the distance between the proximal leaflet and the substrate, reducing detrimental interactions between membrane proteins and the underlying support and creating more physiologically relevant conditions. By providing a flexible, hydrated environment, the polymer cushion enhances membrane protein and lipid mobility, preserves their structural integrity and functionality, and facilitates the study of membrane‐embedded and membrane‐associated proteins under conditions closely mimicking their natural biological context.^[^ ^1^, ^2^, ^25^, ^27^ ^]^

Although polymer cushions improve SLB functionality by minimizing direct interactions with the substrate, they can also introduce unexpected changes to the lipid bilayer's thickness and structural integrity, due to polymer chains embedding within the bilayer.^[^ ^28^, ^29^, ^30^ ^]^ Such variations in bilayer thickness introduced by polymer cushions are of particular interest, as the optimal functioning and localization of many membrane‐embedded proteins depend on the precise alignment of their hydrophobic cores with the hydrophobic region of the lipid bilayer.^[^ ^31^, ^32^, ^33^ ^]^ Therefore, accurate determination of the structural and leaflet‐specific dynamic properties of SLBs, especially in the presence of polymer support, is essential for membrane protein and model membrane studies. A simultaneous assessment of these properties within a single measurement is essential for achieving a comprehensive understanding of SLB behavior and its implications for membrane protein functionality.

Although a variety of techniques, such as those based on X‐ray^[^ ^34^ ^]^ reflectometry or NR,^[^ ^35^, ^36^ ^]^ and contact‐based methods, such as AFM,^[^ ^37^ ^]^ have been employed to elucidate the structural properties of SLB, the experimental investigation of their dynamics in a leaflet‐specific manner remains limited. Methods such as single‐particle tracking,^[^ ^15^, ^38^ ^]^ fluorescence correlation spectroscopy (FCS),^[^ ^16^, ^39^ ^]^ two‐dimensional fluorescence lifetime correlation spectroscopy (2D FLCS),^[^ ^40^ ^]^ and fluorescence recovery after photobleaching (FRAP)^[^ ^41^ ^]^ are routinely used to study the dynamics of lipids and proteins in model membranes, but each have inherent limitations. For instance, single‐particle tracking may not be well‐suited for bilayers or leaflets exhibiting rapid diffusion, as it struggles to capture fast‐moving lipid dynamics.^[^ ^17^ ^]^ Leveraging the selective quenching of fluorescence in the distal leaflet using dynamic dark quenchers in solution, FCS and 2D FLCS can resolve leaflet‐specific dynamics.^[^ ^40^ ^]^ However, this approach requires millimolar concentrations of chemical additives, that can disrupt the lipid's native chemical environment and potentially influence membrane behavior. Furthermore, these techniques generally fall short in providing any structural details alongside the dynamic measurements, limiting their ability to offer a holistic understanding of SLB systems.

In this study, we present a novel methodology that integrates graphene‐induced energy transfer (GIET) with line‐scan fluorescence lifetime correlation spectroscopy (lsFLCS). This approach addresses the limitations of the current state‐of‐the‐art by enabling the simultaneous measurement of structural and dynamic properties in p‐SLBs. Previously, we demonstrated the capability of GIET to measure the thickness of an SLB and the hydration layer thickness with Ångström‐level accuracy as a function of its composition.^[^ ^19^, ^42^, ^43^ ^]^ In another recent study, we combined GIET with FCS to quantify the nanometer‐scale and millisecond‐timescale undulations of model bilayers and organelle membranes.^[^ ^44^ ^]^ Additionally, fluorescence lifetime correlation spectroscopy (FLCS) has been used to quantify microsecond state transitions of a fluorescent protein due to rotational isomerization of an amino acid next to its chromophore.^[^ ^45^, ^46^ ^]^ Here, we advance these methods by applying lsFLCS, which, unlike point excitation used in FCS and 2D FLCS, enables the separation and precise determination of leaflet‐specific lipid diffusion in a calibration‐free and statistically robust manner. This advantage stems from the repeated scanning along a defined line, enabling lsFLCS to probe diffusion over a larger area, thereby improving data statistics. Furthermore, the precisely controlled scanning allows for accurate determination of the excitation focus size without the need for additional calibration experiments, which are required in traditional point FCS.

Our methodology begins by validating the GIET‐lsFLCS approach on solid‐supported lipid bilayers (s‐SLBs) without any polymer support. This combined technique allows us to determine essential parameters, such as bilayer height, thickness, and diffusion coefficients specific to each leaflet of the bilayer. This initial validation forms the foundation for our subsequent investigation into p‐SLBs, focusing on three commonly used polyethylene glycol (PEG) SLBs. These PEG‐based SLBs utilize different strategies to tether or cushion the lipid bilayers with PEG chains.

By applying our method, we comprehensively examine the structure and dynamics of these PEG‐supported bilayers. Our findings highlight the influence of PEG chain presence and arrangement on the bilayer's structural characteristics and the diffusion behavior of the leaflets. This investigation provides insights into the interplay between PEG chains and lipid bilayers, shedding light on the structural and dynamic properties of these complex systems.

## Results and Discussion

**Figure 1:**
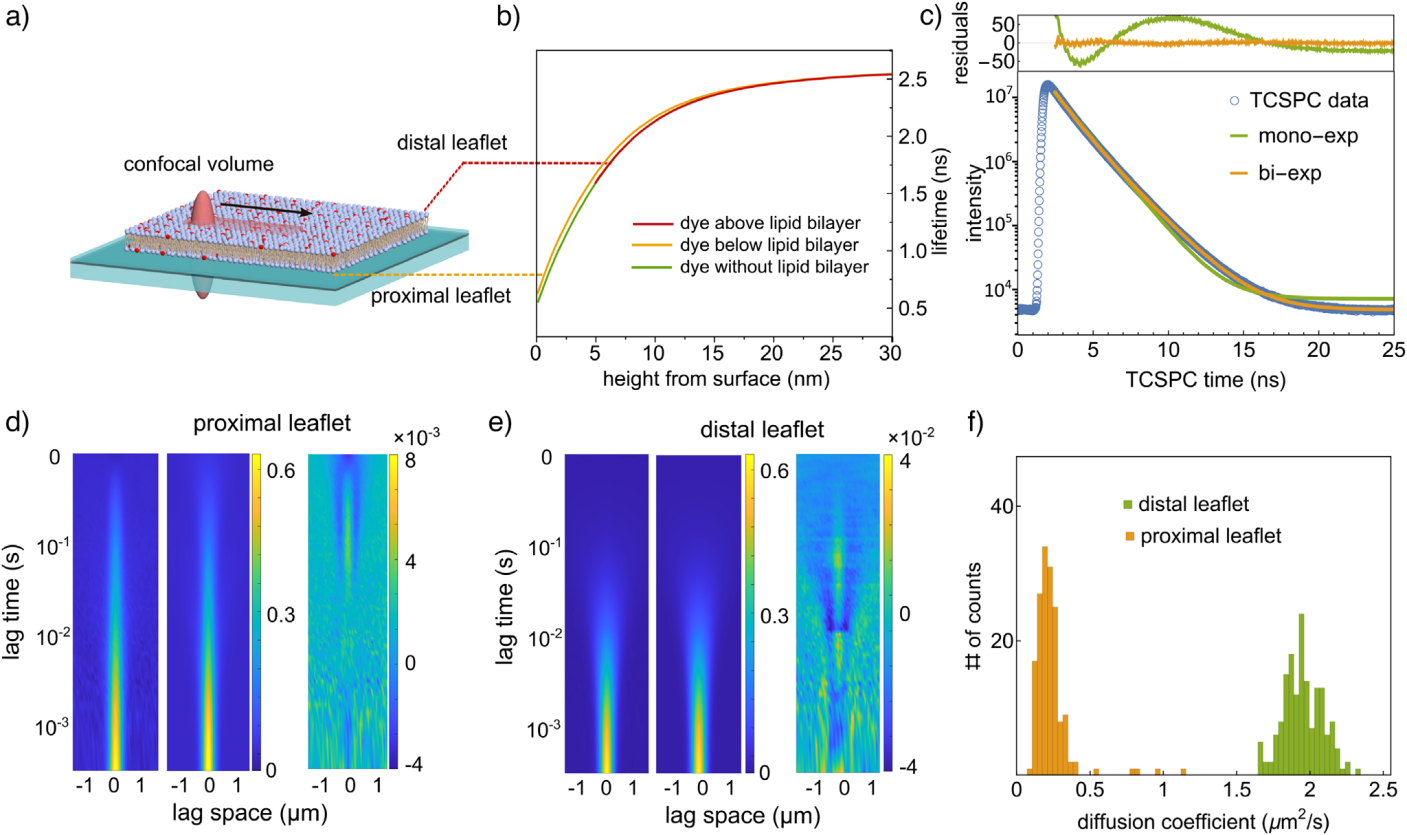
GIET‐lsFLCS measurement on SLB to uncover bilayer thickness and leaflet‐specific diffusion. a) A schematic representation of the experimental setup, illustrating the s‐SLB positioned on a graphene substrate with a 10 nm silica spacer. The schematic details the procedure of scanning a confocal volume linearly and repeatedly at a constant velocity across the membrane.b) The calculated fluorescence lifetime profile for Atto655‐DPPE dye within the SLB, plotted as a function of distance from the surface. The calculation considers a single emitter with an emission wavelength of 680 nm, a lifetime of 2.6 ns, and a quantum yield of 0.36. c) Fluorescence lifetime decay curve obtained from the labeled SLB on the graphene substrate. The data were analyzed using mono‐exponential (green) and bi‐exponential (brown) decay models, with the upper panel displaying the fitting residuals. d) The derived autocorrelation function for the proximal leaflet (left), along with the corresponding fitting results (middle), and the residuals from the fit (right). e) An analogous analysis for the distal leaflet, following the same structure as described in d). f) Histograms depicting the diffusion coefficients for both the proximal and distal leaflets of the SLB (*N* = 161 for each histogram). These values are obtained through bunchwise fitting of the autocorrelation functions, revealing the distinct diffusion dynamics of each leaflet.

The experimental setup used for conducting GIET and lsFLCS measurements on SLBs is depicted in Figure 1a. To validate the methodology, we first applied it to an s‐SLB that did not contain any polymer support. This s‐SLB consisted of pure dioleoylphosphatidylcholine (DOPC) mixed with a low concentration of 1,2‐dipalmitoyl‐sn‐glycero‐3‐phosphoethanolamine (DPPE‐Atto655) as a fluorescently labeled head group. We prepared the SLB, using the vesicle fusion method on a GIET substrate, which consists of a graphene monolayer on top of a standard glass coverslip, topped with a 10 nm thick silica layer. For our experiments, we employed a confocal laser scanning microscope with a rapid galvo scanning unit, pulsed lasers, and a time‐correlated single‐photon counting (TCSPC) unit. The laser excitation focus was repeatedly scanned back and forth across the SLB on top of the GIET substrate along a linear path (path length: 5 µm) at a constant velocity of 0.02 m s^−1^. The collected fluorescence photons were timed relative to their corresponding excitation laser pulses and recorded using the TCSPC unit.

The graphene monolayer (∼ 0.5 cm × 0.5 cm, Figures S1 and S2), sandwiched between a standard glass coverslip and a thin silica spacer of 5 or 10 nm, quenches the fluorescence of dye molecules next to it. This fluorescence quenching, both in intensity and lifetime, is driven by the energy transfer from the excited state of the fluorophore to the electron‐hole pairs in graphene and is highly distance‐dependent.^[^ ^19^, ^42^, ^43^ ^]^ This near‐field coupling between the fluorescent dye molecule and the GIET substrate can be calculated using a semi‐classical electrodynamics model that has been described and validated in previous studies.^[^ ^47^, ^48^, ^49^ ^]^ Within the SLB, the fluorescence lifetime of a dye labeling the lipid head groups in the proximal leaflet, which lies closer to the graphene monolayer, is significantly shorter than that of the same dye on the distal leaflet, located farther away.^[^ ^42^, ^43^ ^]^

We calculated the distance‐dependent fluorescence lifetimes of dye molecules in the bottom leaflet and the top leaflet by considering the refractive index of the media and the lipid bilayer, the thickness and refractive indices in the stratified GIET substrate, the quantum yield, and the emission spectrum of the dye molecules. Here, we approximate the bilayer as 5 nm thick dielectric material with a refractive index of 1.46.^[^ ^50^ ^]^ The resulting distance‐lifetime dependence of dyes in the proximal and distal leaflets is referred to as the GIET calibration curves. Figure 1b shows the GIET calibration curve calculated for DPPE‐Atto655 molecule, which was used in the SLB experiments in this study. This molecule has a mono‐exponential fluorescence lifetime of 2.6 ns and a quantum yield of 0.36 in s‐SLB on top of a pure glass substrate (Figure S3).^[^ ^42^ ^]^ The emission transition dipole orientation of the dye is assumed to be parallel to the plane of the SLB as was shown in previous results.^[^ ^19^, ^42^ ^]^ For the GIET substrate, we adopted a graphene layer thickness of 0.34 nm with a refractive index of = 2.77 + 1.41i (corresponding to an emission wavelength of 680 nm) and set the thickness of the silica (refractive index = 1.46) layer above the graphene to 5 nm, with the half‐space above consisting of water (refractive index = 1.33). Note that we assumed the thickness of the SLB to be 5 nm, approximating its actual thickness, to simplify the GIET calibration curve calculations. Variations of ±1 nm in actual thickness result in lifetime deviations of less than 0.2%, which are negligible compared to the experimental measurement accuracy and can therefore be disregarded.

From the linescan measurements, TCSPC histograms were computed and fitted with a bi‐exponential decay function, as shown in Figure 1c. For statistical analysis, we partitioned the photons collected from each measurement (with more than 10^9^ photons recorded per sample) into smaller sets, each containing 10^7^ photons. We constructed TCSPC curves from these photon datasets and fitted them with a bi‐exponential decay model to obtain lifetime values for each leaflet (see Figures S4 and S5). The fitted decay times are τ_*p*_ = 0.99 ± 0.1 and τ_*d*_ = 1.83 ± 0.2 ns, corresponding to the fluorescence lifetimes of dyes in the proximal and distal leaflets, respectively. Dyes in the proximal leaflet exhibit shorter lifetime values because they are closer to the graphene layer. Using the GIET calibration curve, we converted these lifetime values into height measurements. Based on these decay times, we estimated the thickness of the s‐SLB to be 5.0 ± 0.08 nm and a hydration layer beneath the membrane to be 1.3 ± 0.3 nm, which is consistent with previous atomic force microscopy data reporting head‐group‐to‐head‐group distances of 4.6 ± 0.2 nm.^[^ ^51^ ^]^ It should be noted that the bilayer thickness as determined here is the distance between dye positions in the proximal and distal leaflets. This distance is larger than head‐group‐to‐head‐group distance due to the finite linker length and the size of the dye molecules.

Next, the two mono‐exponential components of this bi‐exponential decay were used to obtain filter functions to calculate lifetime‐specific spatiotemporal fluorescence correlation maps (see Figure S6), as shown in Figure 1d and detailed in the Supporting Information. Briefly, these filter functions constitute an orthonormal basis for fluorescence decay patterns. When applied bin by bin to the fluorescence decay function and summed, the result is either one or zero, depending on whether the filter function matches the given fluorescence decay (or not).^[^ ^46^ ^]^ When applying these filter functions prior to calculating the correlation functions, this leads to two distinct spatiotemporal autocorrelation functions—one for each lifetime component (refer to Experimental Methods, Equation 3, Supporting Information) *g*_*ii*_(*x*, *t*) for *ii* = 1, 2. In Figure 1d,e, the *x*‐axis denotes the spatial lag variable, while the *y*‐axis represents the discrete lag time *t*. These spatiotemporal autocorrelation functions describe the probability of successfully detecting a photon from a molecule at position *x* and time *t* after a photon was detected from the same molecule at position *x* = 0 and time *t* = 0. The dependence of these autocorrelation functions, Figure 1d,e, on the spatial lag variable *x* is determined by the size and shape of the excitation focus, while the temporal correlation decay and broadening with time *t* is due to the lateral diffusion of molecules. As evident from Figure 1e, the decay of the autocorrelation function associated with the longer fluorescence lifetime (at lag space = 0) is significantly faster than that of the autocorrelation function for the shorter fluorescence lifetime, indicating a slower diffusion coefficient of lipids in the proximal leaflet.

Fitting each autocorrelation function using a two‐dimensional scan‐and‐diffusion model allowed us to extract the diffusion coefficients for both leaflets (Figure 1d,e).^[^ ^52^ ^]^ This analysis provides insights into the distinct diffusion behaviors of the proximal and distal leaflets in the SLB system. We applied the same statistical approach as described above to generate spatiotemporal correlation maps from photon sets with 10^7^ photons each. We determined the diffusion coefficient of labeled lipids in the distal leaflet to be 1.95 ± 0.12 µm^2^ s^−1^, which is over eight times faster than that of the proximal leaflet, determined to be 0.23 ± 0.06 µm^2^ s^−1^ (Figure 1f). This significant difference in diffusion rates highlights the unique dynamics of lipid molecules in each leaflet and underscores the substantial impact of substrate interactions on the proximal leaflet, severely hindering the lipid diffusion compared to the distal leaflet.

Having successfully demonstrated GIET‐lsFLCS for measuring bilayer thickness and separating leaflet‐specific diffusion in s‐SLBs, next, we applied our methodology to investigate various p‐SLB systems. Our study focused on three distinct types of PEG‐modified lipid bilayers, which have been extensively used in previous research:^[^ ^1^, ^25^, ^27^ ^]^

**Figure 2:**
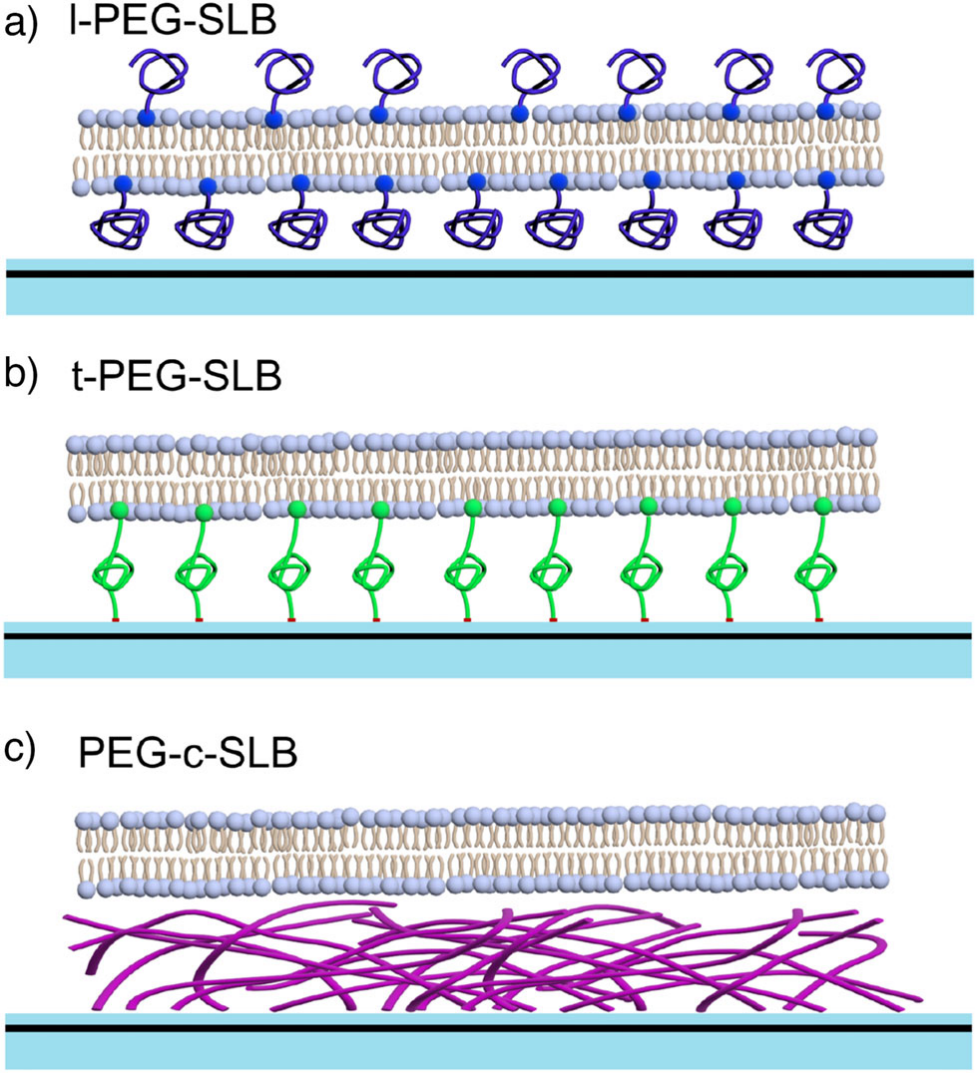
Scheme of the three types of p‐SLBs. a) l‐PEG‐SLB. b) t‐PEG‐SLB. c) PEG‐c‐SLB.

(1) Lipid PEGylated SLB (l‐PEG‐SLB) (Figure 2a):^[^ ^53^, ^54^, ^55^ ^]^ This system was created by fusing PEGylated liposomes on top of a GIET substrate. The liposomes consisted of DOPC and a proportion of 1,2‐distearoyl‐sn‐glycero‐3‐phosphoethanolamineN‐[methoxy(polyethylene glycol)‐2000] (or PEG2000/PE), where the linear PEG chains are attached directly to the lipid head groups. Consequently, a PEG layer is formed around the membrane surface, but the individual PEG chains are not covalently anchored to the substrate. We analyzed l‐PEG‐SLBs with varying percentages of PEG2000‐PE to observe how the PEG density impacts the membrane properties.
(2) Tethered PEGylated SLB (t‐PEG‐SLB) (Figure 2b): In this system, the PEG chains were covalently tethered to the silica surface on top of the GIET substrate via NH_2_‐PEG2000‐PE. Unlike l‐PEG‐SLB, the terminal amine groups of the PEG chains in t‐PEG‐SLB are chemically immobilized on the surface,^[^ ^56^, ^57^ ^]^ providing a more stable attachment and potentially altering membrane behavior.
(3) PEG‐Cushioned SLB (PEG‐c‐SLB) (Figure 2c):^[^ ^13^ ^]^ This bilayer was created by depositing lipid vesicles on top of a GIET substrate modified with a PEG brush. The brush was created by covalently attaching one end of amine‐functionalized PEG(2000) polymers to the substrate, while the other end was coupled to palmitic acid moieties. This configuration provides a soft, cushioned interface between the bilayer and the substrate, reducing direct lipid–substrate interactions.

It is important to note that all three SLB systems use PEG chains with identical molecular weights (MW = 2000). Our analysis aims to compare three key parameters across these p‐SLBs and s‐SLB: membrane height from the substrate (the distance between the proximal leaflet and the silica surface), membrane thickness, and leaflet‐specific diffusion coefficients.

**Figure 3:**
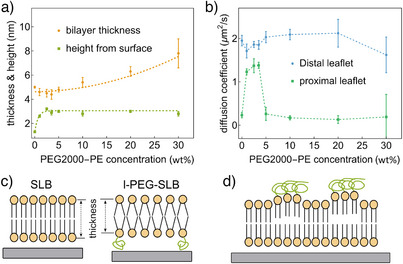
Structure and dynamics of l‐PEG‐SLBs with varying the PEG2000‐PE concentrations. a) The variations in thickness and height of l‐PEG‐SLBs as a function of different PEG2000‐PE concentrations. For better visualization, least‐square fits of parabolic curves to the data are also shown. These fits shall help to follow the general trend of thickness/height‐vs‐PEG concentration dependence, but do not suggest an actual physical square PEG‐concentration law of this dependence. b) The diffusion coefficients of the proximal leaflet and distal leaflet as a function of PEG2000‐PE concentrations. The error bar is standard deviations. Numerical values are listed in Table S1. c) Schematic illustrating the thickness reduction effect caused by the free‐standing geometry. d) Schematic depicting the potential local decoupling effect resulting from polymer clustering.

The structural and dynamic properties of l‐PEG‐SLBs are significantly influenced by the concentration of PEG2000‐PE within the bilayer.^[^ ^54^ ^]^ To optimize this system, we first identified the ideal PEG2000‐PE concentration by characterizing the structure and diffusion of l‐PEG‐SLBs with different PEG2000‐PE concentrations (Figure S7). Figure 3a illustrates the relationship between the thickness and the proximal leaflet height of the l‐PEG‐SLB and varying PEG2000‐PE concentrations. Interestingly, we found that the membrane heights of l‐PEG‐SLBs consistently reached approximately 3 nm across all PEG2000‐PE concentrations. Our results align with prior studies, which reported an estimated height of around 4 nm for l‐PEG‐SLBs containing approximately 15 wt% PEG2000‐PE.^[^ ^53^ ^]^ In our experiments, we explored a PEG2000‐PE concentration range from 0 wt% to 30 wt%, as concentrations beyond 30 wt% prevented vesicle rupture on the surface (Figure S8). For comparison, the s‐SLB exhibited a hydration layer between the proximal leaflet and the substrate measuring 1.3 ± 0.1 nm.

The uniform membrane heights observed across different PEG2000‐PE concentrations in l‐PEG‐SLBs present an intriguing anomaly, deviating from conventional expectations. Typically, when PEG chains are sparsely grafted, the distance between grafting points is much larger than the polymer's radius of gyration, resulting in minimal lateral interactions and leading the chains to adopt isolated, coil‐like conformations characteristic of the “mushroom regime.” As the chain density increases beyond a critical threshold, lateral interactions become significant, forcing the chains to stretch outward perpendicularly from the membrane surface, thereby transitioning into the “brush” regime. This transition should result in increased membrane height with higher PEG density.^[^ ^58^, ^59^ ^]^ Previous research estimated that for PEG2000‐PE, this transition concentration is around 1.4 mol% (approximately 5 wt%).^[^ ^54^, ^60^ ^]^ The lack of height variation in our system may be due to the noncovalent attachment of the PEG chains to the substrate.

In l‐PEG‐SLBs, where PEG chains are grafted onto lipid molecules, the lateral mobility of both PEG2000‐PE and lipids within the flexible membrane could counteract the forces required for the chains to adopt a vertical conformation. Notably, previous studies have shown that even at PEG2000‐PE concentrations as high as 5 mol% (roughly 20 wt%), the diffusion of PEG2000‐PE molecules is reduced by only half.^[^ ^54^ ^]^ This behavior challenges conventional models of PEG chain interactions, suggesting the presence of additional influencing factors, such as specific interactions between PEG chains, lipids, and the substrate.

Although membrane heights remained consistent across different PEG2000‐PE concentrations, lower PEG‐PE content (ranging from 1 wt% to 3.5 wt%) led to a reduced bilayer thickness (Figure 3a). For example, the thickness of PEG2000‐PE with 3.5 wt% (4.4 ± 0.4 nm) is ∼ 0.6 nm thinner than the s‐SLB (5.0 ± 0.08 nm). This effect may result from changes in lipid packing density,^[^ ^55^ ^]^ which could lead to the formation of “free‐standing” lipids characteristic of the “mushroom” regime (Figure 3c). Additionally, at high PEG chain densities, the distribution of PEG chains on both sides of the bilayer might induce a decoupling effect between the two leaflets,^[^ ^61^, ^62^ ^]^ contributing to the observed increase in bilayer thickness for the 20 wt% and 30 wt% PEG2000‐PE samples (Figure 3d).

Further, we determined the diffusion coefficients for the proximal (*D*_proximal_) and distal (*D*_distal_) leaflets across all l‐PEG‐SLBs (Figure 3b). Notably, in all samples, the distal leaflets exhibited nearly identical diffusion rates, consistently faster than the proximal leaflets. However, distinct behaviors were observed in the proximal leaflets concerning PEG2000‐PE concentration. In the range of 1 wt% to 3.5 wt% PEG2000‐PE, the proximal leaflets displayed rapid diffusion, with similar diffusion coefficients (*D*_distal_ ∼1.4 µm^2^ s^−1^). Conversely, in l‐PEG‐SLBs with higher PEG2000‐PE concentrations (5 wt%–20 wt%), *D*_proximal_ values were significantly lower, akin to those observed in s‐SLBs (∼ 0.2 µm^2^ s^−1^).

Interestingly, the l‐PEG‐SLB membrane containing 30 wt% PEG2000‐PE exhibited reduced lipid mobility in the distal leaflet compared to other samples. The observed variations in diffusion within the proximal leaflet were associated with changes in membrane thickness at different PEG2000‐PE concentrations. The slower diffusion observed in l‐PEG‐SLBs with higher PEG2000‐PE content suggests strong interactions between proximal lipids and the PEG chains. In contrast, l‐PEG‐SLBs with lower PEG2000‐PE concentrations (ranging from 1 wt% to 3.5 wt%) showed significantly weaker interactions with the polymer layer. Our findings of reduced mobility in l‐PEG‐SLBs are consistent with previous studies using FRAP measurements.^[^ ^54^, ^63^ ^]^ Based on these observations, we selected the l‐PEG‐SLB with 2.5 wt% PEG‐PE (referred to as 2.5l‐PEG‐SLB) for further comparison with other SLBs where PEG chains are anchored to the surface (Figure 2).

**Figure 4:**
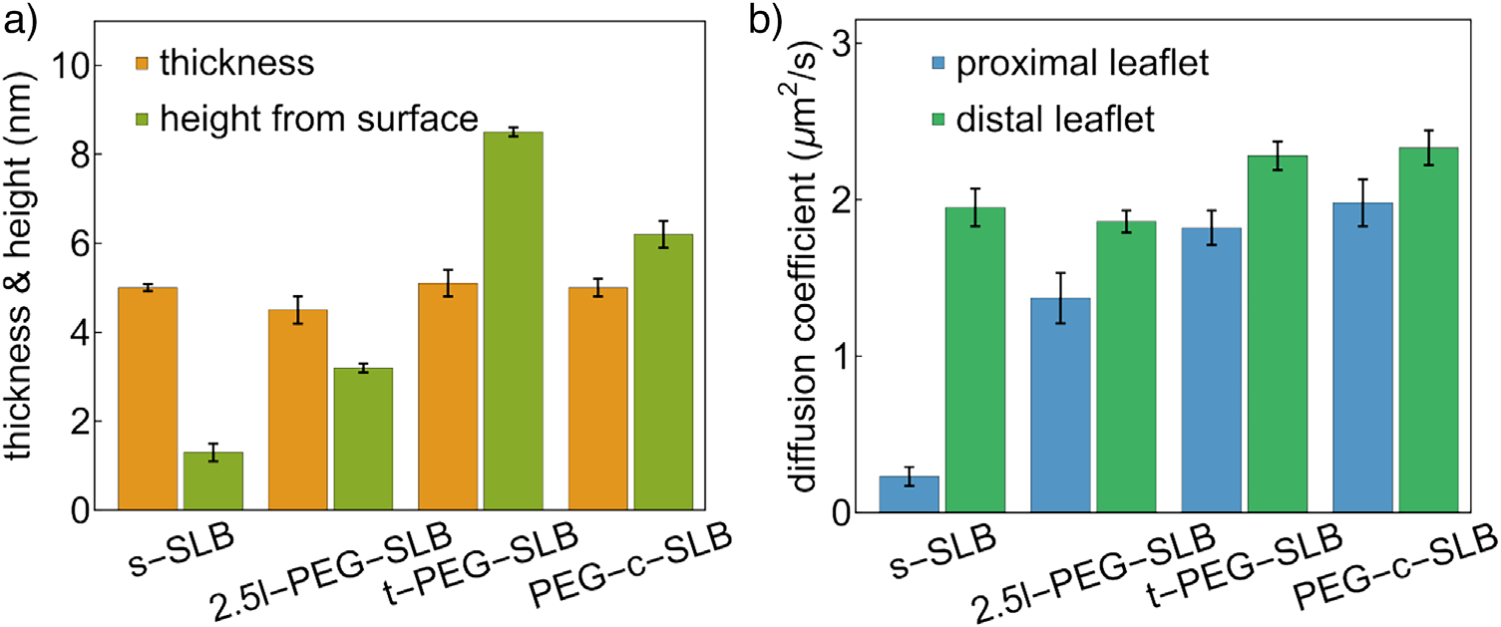
Structure and dynamics of different SLBs. a) The thickness and height of four different SLBs. b) The diffusion coefficients of the proximal and distal leaflets of the four SLBs. Error bars represent standard deviations. Numerical values are provided in Table S1.

We then applied GIET‐lsFLCS to comprehensively analyze the structural properties and leaflet‐specific diffusion of t‐PEG‐SLB and PEG‐c‐SLB membranes, facilitating direct comparison with other SLBs (Figure 4). As shown in Figure 4a, both the t‐PEG‐SLB and PEG‐c‐SLB exhibited membrane thicknesses similar to the SLB without polymer. However, their heights were significantly greater than those of s‐SLB and 2.5l‐PEG‐SLB: the proximal leaflet t‐PEG‐SLB reached a height of 8.5 ± 0.1 nm, and the PEG‐c‐SLB measured 6.2 ± 0.3 nm. This increased height is attributed to the presence of PEG chains grafted to the solid surface, creating a “brush” regime. The observed height difference between l‐PEG‐SLB and t‐PEG‐SLB aligns with previous findings using neutron and X‐ray reflectivity measurements reported by Watkins et al.^[^ ^64^ ^]^ Their study demonstrated that the membrane height of l‐PEG‐SLB is similar to that of s‐SLB, while t‐PEG‐SLB is elevated approximately 7 nm above the surface when the terminal ends of the lipopolymers are anchored to the substrate. They attributed the absence of a height increase in l‐PEG‐SLB to the redistribution of all lipopolymers to the upper leaflet, resulting in no polymer cushion beneath the bilayer. In contrast, our measurements indicate a 1 nm height increase and enhanced diffusion in l‐PEG‐SLB at low lipopolymers concentration, suggesting the presence of some lipopolymers beneath the bilayer.

In terms of leaflet‐specific diffusion rates (Figure 4b), both the t‐PEG‐SLB and PEG‐c‐SLB displayed considerably faster diffusion compared to the l‐PEG‐SLB and s‐SLB in both proximal and distal leaflets. The relatively slow diffusion observed in the distal leaflet of the l‐PEG‐SLB could be attributed to interactions between the distal lipids and PEG(2000) chains on top of the bilayer. Although all membranes comprised DOPC lipids, the differences in *D*_distal_ values among the SLBs indicate that the distal leaflet's behavior is not independent. Rather, there is significant interleaflet mixing and coupling, wherein the properties of one leaflet significantly influence those of the opposing leaflet.^[^ ^12^, ^22^, ^65^, ^66^ ^]^ Molecular dynamics simulations have confirmed substantial interleaflet mixing, estimating approximately 71% interleaflet mixing in DOPC bilayers.^[^ ^22^ ^]^ This interleaflet mixing significantly impacts the mobility of the distal leaflet, which is influenced by the dynamics of the proximal leaflet. Consequently, the faster diffusion observed in the distal leaflet of t‐PEG‐SLB and PEG‐c‐SLB can be attributed to the rapid diffusion in their respective proximal leaflets. In contrast, in the 2.5l‐PEG‐SLB, the presence of PEG chains interacting with the distal leaflet slows down the lipid diffusion.

Our comparisons (Figure 4 and Table S1) indicate that t‐PEG‐SLB and PEG‐c‐SLB membranes are superior platforms for biophysical applications due to their greater separation from the substrate and faster diffusion rates. However, their preparation is labor‐intensive and time‐consuming. On the other hand, l‐PEG‐SLBs are easier to prepare, making them a popular choice for studies that aim to mimic the plasma membrane.^[^ ^53^, ^55^, ^67^, ^68^, ^69^, ^70^ ^]^ Caution should be exercised when using l‐PEG‐SLBs, particularly with PEG2000‐PE concentrations ranging from 1.5 mol% to 5 mol% (equivalent to 5 wt% to 20 wt%), as these concentrations can yield diffusion rates comparable to those of s‐SLBs. Moreover, the height provided by l‐PEG‐SLBs using PEG2000‐PE is limited to 3 nm, which may not be suitable for certain transmembrane proteins or applications requiring greater membrane height.

## Conclusion

In summary, our study introduces GIET‐lsFLCS as a robust and versatile method for characterizing both the structural and dynamic properties of SLBs, offering detailed insights that are critical for advancing membrane and membrane protein biochemistry. By examining three commonly used p‐SLBs with distinct PEG chain conformations, we demonstrated how polymer architecture significantly influences membrane height and lipid diffusion, thereby elucidating key design considerations for SLB‐based biotechnological and medical applications. Furthermore, our investigation into leaflet‐specific lipid diffusion provides insights into interleaflet coupling mechanisms, enriching our understanding of lipid bilayer physics and the behavior of asymmetric bilayers. This information not only deepens the fundamental knowledge of membrane biophysics but also paves the way for studying lipid interactions in complex biological systems. Importantly, the versatility of GIET‐lsFLCS positions it as a promising tool for broader membrane research, enabling the exploration of lipid bilayer behavior under various conditions and facilitating studies on interactions with various biological molecules.

## Conflict of Interests

The authors declare no conflict of interest.

## Supporting information

Supporting Information

## Data Availability

The data that support the findings of this study are available from the corresponding author upon reasonable request.
