# Supplementary material for: Leaflet‐Specific Structure and Dynamics of Solid and Polymer Supported Lipid Bilayers
Source: Angew Chem Int Ed Engl. 2025 Apr 7;64(21):e202423784. doi: 10.1002/anie.202423784 (PMC12087848; doi:10.1002/anie.202423784)
Supplement: Supplementary file 1 — Supporting Information [file ANIE-64-e202423784-s001.pdf]

Supporting information:

Leaflet-specific Structure and Dynamics of Solid and Polymer Supported Lipid Bilayers

Narain Karedla,[a,b,c] Falk Schneider,[d,e] Jörg Enderlein,[*,a,f] and Tao Chen,[*,a]

[a]Third Institute of Physics – Biophysics, Georg August University, Friedrich-Hund-Platz 1, 37077 Göttingen, Germany
[b]The Rosalind Franklin Institute, Harwell Campus, Didcot, OX11 0FA, United Kingdom
[c]Kennedy Institute of Rheumatology, University of Oxford, Roosevelt Drive, Oxford, OX3 7LF, United Kingdom
[d]Translational Imaging Center, University of Southern California, Los Angeles, California, 90089, United States of America
[e]Biomedical Sciences, Warwick Medical School, University of Warwick, Coventry, United Kingdom
[f]Cluster of Excellence "Multiscale Bioimaging: from Molecular Machines to Networks of Excitable Cells" (MBExC), Universitätsmedizin Göttingen, Robert-Koch-Str. 40, 37075 Göttingen, Germany

E-mail: tao.chen@phys.uni-goettingen.de; jenderl@gwdg.de

Materials

The lipids, including 1,2-dioleoyl-sn-glycero-3-phosphocholine (DOPC), 1,2-distearoyl-sn-glycero-3-phosphoethanolamine-N-[amino(polyethylene glycol)-2000] (ammonium salt) (DSPE-PEG2000-Amine), and 1,2-distearoyl-sn-glycero-3-phosphoethanolamine-N-[methoxy(polyethylene glycol)-2000] (ammonium salt) (PEG2000-PE), were procured from Avanti Polar Lipids. Atto655 dye-labeled 1,2-dipalmitoyl-sn-glycero-3-phosphoethanolamine (DPPE-atto655) was obtained from ATTO-TEC GmbH. Poly(ethylene glycol) diamine (average Mn: 2000; PEG-diamine), palmitic acid, (3-Glycidyloxypropyl)trimethoxysilane (GOPS), and N,N′-Diisopropylcarbodiimide (DIC) were purchased from Sigma-Aldrich.

Methods

Measurement setup: GIET-lsFLCS measurements were conducted using a custom-built confocal microscope equipped with a multichannel picosecond event timer (HydraHarp 400, PicoQuant GmbH) and a fast scanner (FLIMbee Galvo scanner, PicoQuant). A pulsed diode laser emitting at a wavelength of λ_{exc} = 640 nm (LDH-D-C 640, PicoQuant), operating with a pulse width of 50 ps FWHM and a repetition rate of 40 MHZ, served as the excitation source. In the excitation path, a clean-up filter (LD01-640/8 Semrock) was employed. The light beam was collimated with an infinity-corrected 4x objective (UPISApo 4X, Olympus) to a 12 mm diameter and then directed by a dichroic mirror (Di01-R405/488/561/635, Semrock) towards a high numerical aperture objective (UAPON 100X, oil, 1.49 N.A., Olympus). Fluorescence light was focused through a pinhole of 100 µm diameter and then refocused onto an avalanche photodiode (τ-SPAD, PicoQuant). For spectral filtering, a long-pass filter (BLP01-647R-25, Semrock) and a band-pass filter (Brightline HC692/40, Semrock) were placed before the pinhole and the detector, respectively. During a lsFLCS measurement, the excitation focus that was positioned on the GIET substrate interface was repetitively scanned along a line of 5 µm length, with 100 scan positions (pixels) and 2.5 µs dwell time per pixel. The total acquisition time for all GIET-lsFLCS measurements was 2 hours at 2 kHz line frequency.

Graphene substrate preparation: We fabricated monolayer graphene-coated coverslips using the transfer method according to the manufacturer's guidelines (Easy Transfer, Graphenea). Subsequently, these coverslips were coated with SiO_2 spacers, with a thickness of 10 nm for l-PEG-SLB, and of 5 nm for t-PEG-SLB and PEG-c-SLB. SiO_2 deposition was performed via vacuum evaporation (Univex 350, Leybold) at the slowest deposition rate of 0.1 nm/s. Precise control of the spacer layer thickness was achieved using an oscillating quartz crystal monitor, which continuously tracked the thickness throughout the deposition process. We have performed additional AFM measurements to estimate the quality of GIET substrates covered with either with 5 nm SiO_2 or 10-nm SiO_2, and we found that the roughness of the 5-nm SiO2 surface is 1.2 nm, and that of the 10-nm SiO2 surface is 0.8 for (mean square root roughness) showing that the surface is very uniform.

PEG-modified substrate preparation: The preparation of the PEG-cushioned substrate followed a previously reported procedure.[1] Initially, the graphene/SiO_2 coverslip underwent a 30-second plasma treatment, followed by immersion in a 20 mM GOPS (3glycidoxypropyltrimethoxysilane) ethanol solution for 1 hour. The GOPS-modified coverslip was then sequentially rinsed with ethanol, isopropanol, and DMSO. Subsequently, a solution containing 0.5 M palmitic acid and DIC (0.646 M) in DMSO was added to the coverslip and incubated for 45 minutes at room temperature. The coverslip was then rinsed successively with DMSO, ethanol, and H_2O, followed by drying using a stream of N_2.

We prepared the PEG-t-SLB following a previously established method with slight modifications.[2] Initially, DSPE-PEG2000-Amine, a PEGylated lipid, was vacuum-dried for 1.5 hours at 30°C and then dissolved in DMSO at a final concentration of 1 mg/mL. This solution was applied to the GOPS-modified graphene/SiO_2

substrate and incubated at 50°C for 12 hours before cooling to room temperature. To remove any unbound lipids, the substrate was sequentially rinsed with DMSO, isopropanol, and water. It is crucial to maintain the substrates in a wet state throughout the process, and they should be promptly used for preparing the bilayer sample.

Vesicle preparation: Small unilamellar vesicles (SUVs) were prepared using the extrusion method. Initially, a mixture consisting of 60 µL of 10 mg/mL mixture in chloroform containing DOPC, PEG2000-PE at varying concentrations ranging from 0 wt% to 40 wt% (0 wt%, 1 wt%, 2.5 wt%, 3.5 wt%, 5 wt%, 10 wt%, 20 wt%, 30 wt%, 40 wt%) was combined with 1 µL of 0.01 mg/mL DPPE-Atto655. This mixture was dried under vacuum at 30 °C for 1.5 h to eliminate the chloroform. The resulting lipid film was re-suspended in 500 µL of Tris-Cl buffer (20 mM Tris-Cl, 100 mM NaCl, 10 mM $CaCl_2$, pH 7.4) using an ultrasonic bath for 5 min, followed by mixing (Thermomixer Comfort, Eppendorf) at 30°C for 1 h. Subsequently, the solution was extruded through a polycarbonate filter (Whatman) with a pore diameter of 100 nm for 15 cycles.

Giant unilamellar vesicles (GUVs) were prepared using the electroformation method.[3,4] First, 100 µL of a 10 mg/mL DOPC chloroform solution containing 0.02 wt% DPPE-Atto655 was deposited onto an electrode plate and dried under vacuum at 30°C for 3 hours. The dried lipid film was then placed in a custom-made chamber filled with 500 µL of a 300 mM sucrose solution. An alternating current (AC) with a peak-to-peak voltage of 1.6 V at 15 Hz was applied to the chamber for 3 hours, followed by a reduction to 8 Hz for an additional 30 minutes to facilitate the electroformation process. Afterward, 500 µL of Tris-Cl buffer was added to the chamber to collect the GUVs. The vesicle solutions were stored at 4°C and used within 3 days of preparation.

SLB preparation: SLB were formed via the vesicle fusion method. To create s-SLB and l-PEG-SLB, a SUV solution with varying amount of PEG2000-PE was deposited onto the graphene/SiO_2 surface and allowed to incubate for 1 h at room temperature. Subsequently, the surface was thoroughly washed with copious buffer. The sample was stored at 4°C overnight for further measurement. For preparing the t-PEG-SLB and PEG-t-SLB, the stock GUV solution was diluted 50-fold with Tris-Cl buffer. This diluted solution was then deposited onto the PEG-modified or lipid-tethered graphene/SiO_2 surface. After incubating for 10 min at room temperature, the solution was replaced with fresh Tris-Cl buffer.

Lifetime fitting: Each detected photon was recorded with two-time tags, the microtime, representing the arrival time of photons with respect to the last preceding excitation laser pulse, and macro-times, representing the arrival time of photons from the start of the experiment on a coarse-grained time-scale (time increments equal to the repetition period of the laser pulsing). From the micro-times, a time-correlated single-photon counting (TCSPC) histogram can be calculated. In GIET experiments on SLBs containing lipids with their head-groups labeled with dyes exhibiting a mono-exponential fluorescence decay, the TCSPC histogram is fitted with a bi-exponential decay curve where the two decay components correspond to dyes in the proximal and distal leaflets:

$$I_j = \sum_{i=1}^{\mathrm{N}} \frac{a_i}{\tau_i} e^{-j\delta/\tau_i} + b_j \qquad (1)$$

Here, j represents the number of a TCSPC time bin and δ is its bin width, a_i is the total fluorescence amplitude for each decay, and τ_i is the fluorescence decay time of the ith component, $i \in \{1,2,\ldots,\mathrm{N}\}$, b_j represents an offset primarily arising from dark counts and other background sources. A minimum of 1×10^9 counts were recorded for a single SLB measurement.

Conversion of lifetime values to distance values: The theoretical background and technical details for converting fluorescence lifetime values to substrate-fluorophore distance values in GIET have been extensively described in prior publications.[4,5] Briefly, one calculates the fluorescence lifetime $\tau_f(\vartheta, z_0)$ of a dye molecule as a function of its distance z_0 from the surface and its orientation angle ϑ relative to the

surface normal using a semiclassical theory of fluorescence emission based on Maxwell's equations. This computation requires prior knowledge of the lifetime τ_0 and the quantum yield ϕ of the dye without graphene. For our experimental studies, we utilized the dye Atto655 tagged to 1,2-dipalmitoyl-snglycero-3-phosphoethanolamine (DPPE; ATTO-TEC, cat. no. AD 655-151), for which the lifetime and quantum yield had been determined previously as τ_0 = 2.6 ns and ϕ = 0.36.[4] Furthermore, previous work has demonstrated that the preferential orientation of the dye molecules is parallel to the SLB plane due to interaction between the dye and the lipid head groups. Using the calculated GIET calibration curves $\tau_f(\vartheta,z_0)$, the heights of the two bilayer leaflets were determined based on the fitted fluorescence lifetime values. All calculations were performed using a publicly available MIET-GUI software[4,5] (see https://projects.gwdg.de/projects/miet).

Line-scan fluorescence-lifetime spatiotemporal correlation (lsFLCS): The calculation of lifetime-specific spatiotemporal auto- and cross-correlations is based on fluorescence lifetime correlation spectroscopy (FLCS). The core principle of FLCS can be described as an unmixing of the fluorescence signal using fluorescence lifetime information (similar to spectral unmixing using spectral informtaion). The two mono-exponential components of the bi-exponential decay are used to generate filter functions $f_j^{(i)}, i \in \{1,2,\dots,\mathrm{N}\}$, as described in detail in previous publications (see also Figure S5 in Supporting Information).[6,7] These filter functions are then used to weight each recorded photon based on its TCSPC channel *j* (micro-time), after which second-order lifetime-specific spatiotemporal fluorescence correlation curves are calculated as

$$g_{ii'}(x,t) = \sum_{j=1}^{L} \sum_{j'=1}^{L} f_j^{(i)} f_{j'}^{(i')} \langle I_j(x_0,t_0) I_{j'}(x_0+x,t_0+t)\rangle_{x_0,t_0} \qquad (2)$$

where the angular brackets ⟨⟩ denote averaging over all positions x_0 and times t_0, and *L* is the number of TCSPC channels. The times t_0 and *t* increment in discrete time steps equal to the scan time of one line scan. The resulting spatiotemporal correlation function $g_{ii}'(x,t)$ is proportional to the probability of detecting a photon from species i' at time $t + t_0$ and position $x_0 + x$ if there was a photon detection from species *i* at time t_0 and position x_0.

lsFLCS fitting: Assuming that the scan pixel size is significantly smaller than the waist w_0 of the Gaussian excitation focus ("2D detection volume"), the spatiotemporal correlation for a single diffusing species in the focal plane is given by

$$g(x,t) = \frac{1}{c\pi w_0^2} exp[- \cdot [1 + \frac{4D}{w_0^2}(t + \frac{x}{v})]^{-1} + g_\infty \qquad (3)$$

where *v* is the scan speed, *x* is the spatial coordinate, *t* is the correlation lag time, *c* is the concentration of molecules, and *D* is the diffusion coefficient.[8] The function $g(x,0)$ represents the purely *spatial* autocorrelation along one line-scan, and the function $g(0,t)$ represents the purely *temporal* autocorrelation at a fixed position along the scan, providing insight into the molecular dynamics over time. Experimentally determined spatiotemporal correlations are fitted with (3) by minimizing the least squares error. For estimating fit value uncertainties, we calculated the spatiotemporal correlations for subsequent bunches of 10 million consecutive photons and fitted the diffusion coefficient for each bunch.

Supplementary Figures

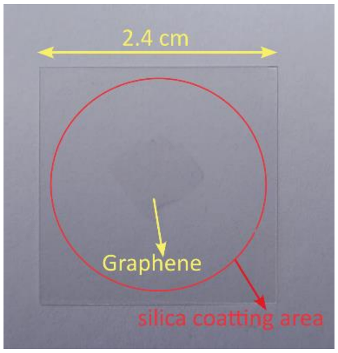

Figure S1. Photo of the GIET substrate.

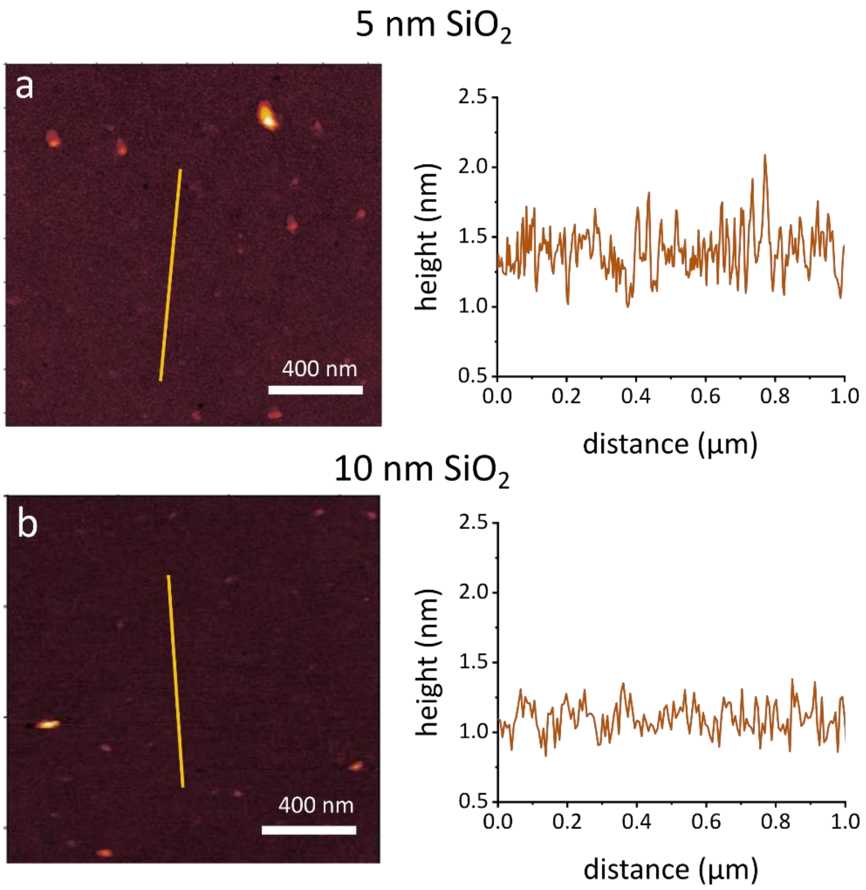

Figure S2. (a, b) Atomic force image (AFM) and the corresponding surface roughness profiles of GIET substrate with 5-nm SiO_2 (a) and 10-nm SiO_2 (b). We obtained root-mean-square values of roughness as 1.2 nm for 5-nm SiO_2 substrate and 0.8 nm for 10-nm SiO_2 substrate, respectively. These surface characterizations affirm that the SiO_2-coated substrate exhibits exceptional smoothness and uniformity.

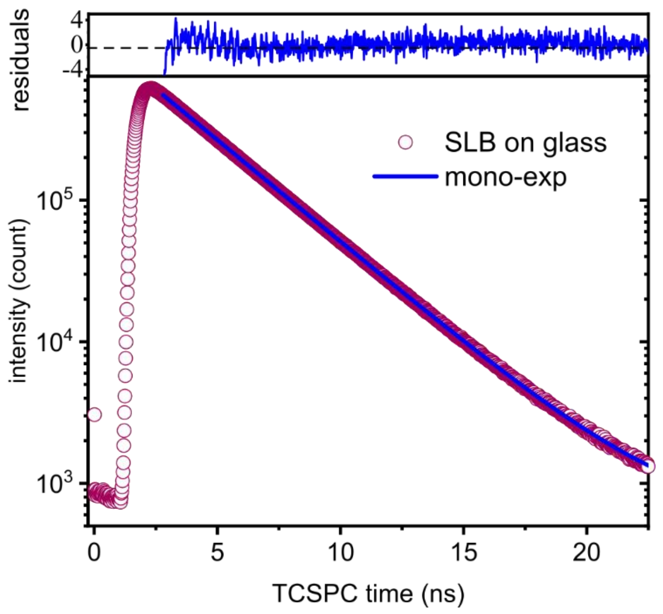

Figure S3. TCSPC histogram and fit for Atto655-DPPE in DOPC SLB on glass substrate. The decay is fitted with single exponential confirming the origin of biexponential behavior only in case of GIET experiments where each lifetime value corresponds to fluorophores in respective leaflets of the SLB.

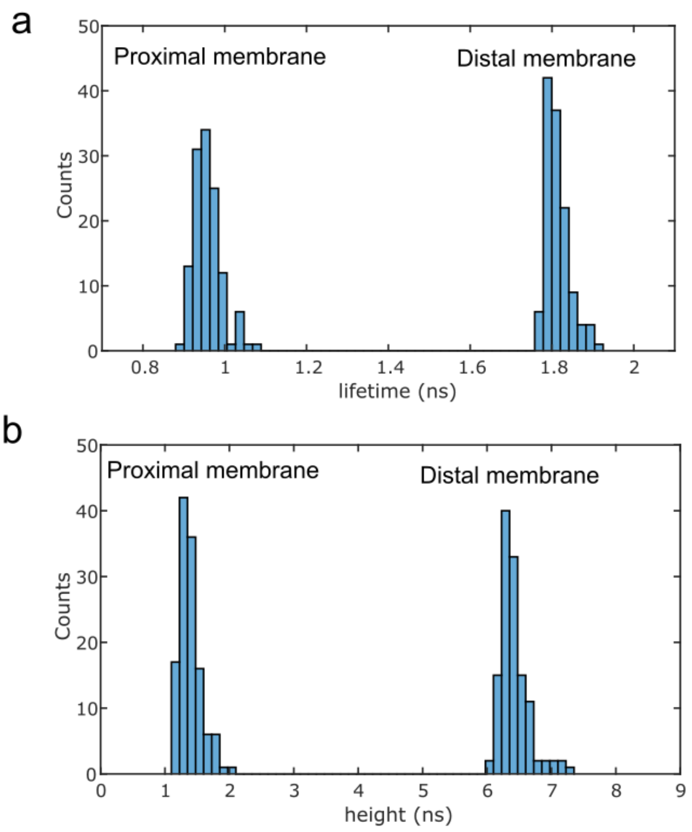

Figure S4. (a) Histograms for short and long lifetime values for DOPC SLB without polymer. (b) The corresponding height values.

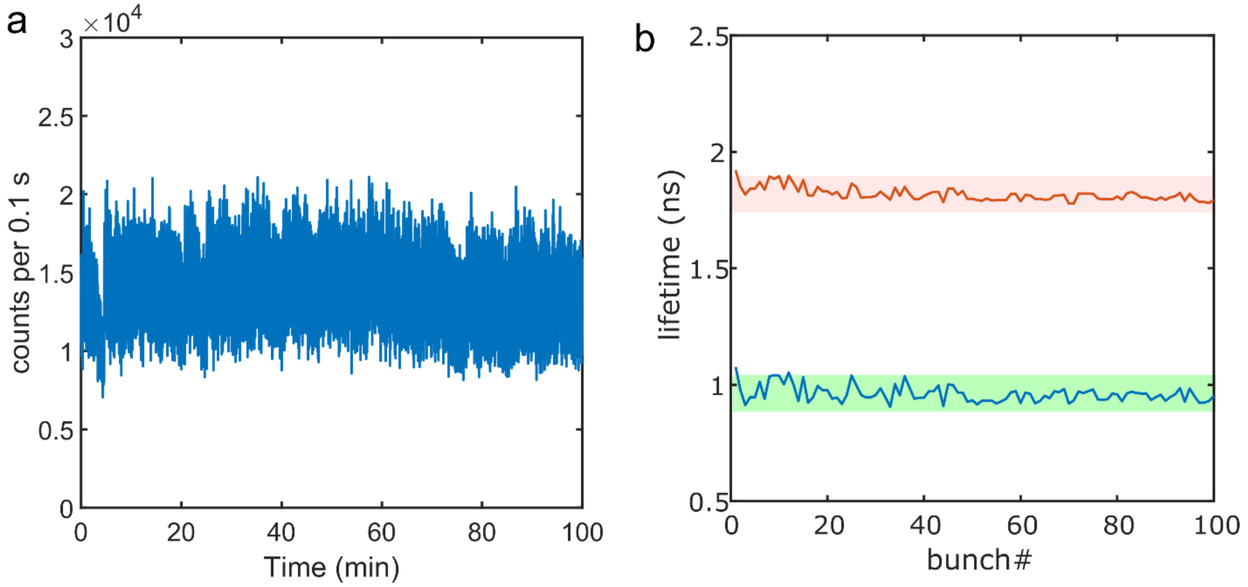

Figure S5. This figure shows that there is no photobleaching effect during the line-scanning measurement and on the two lifetime components. The data is measured from the SLB without polymer (a) The intensity time trace. (b) The lifetime values for each bunch comprising the 10 million photons.

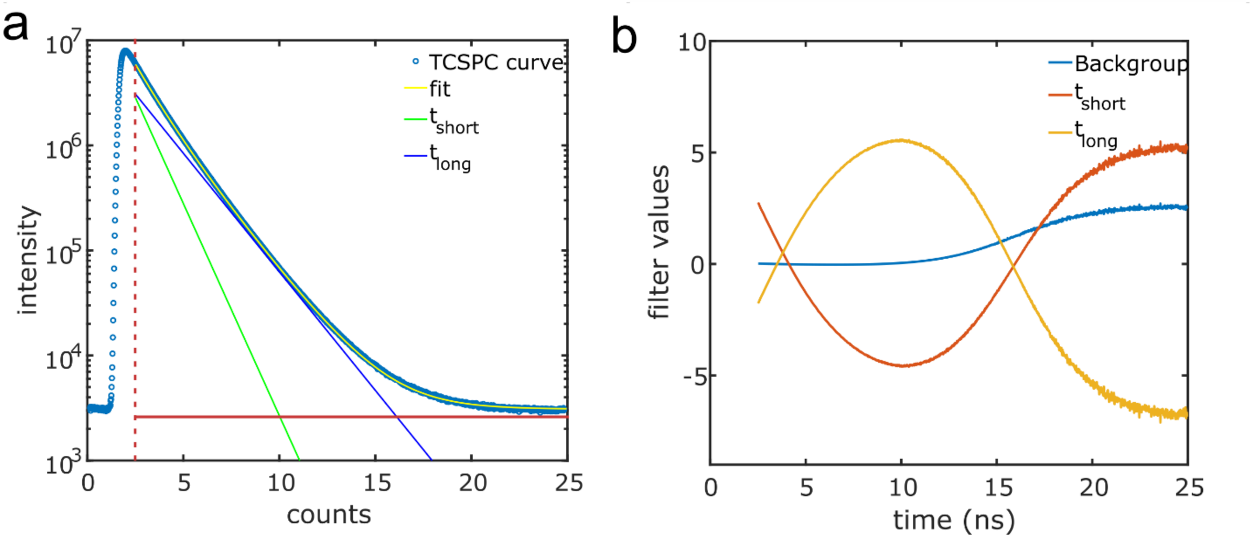

Figure S6. The left figure shows the TCSPC data together with the two lifetime components and a constant offset (solid red line) representing the background signal. The right figure shows the calculated filter using the three components.

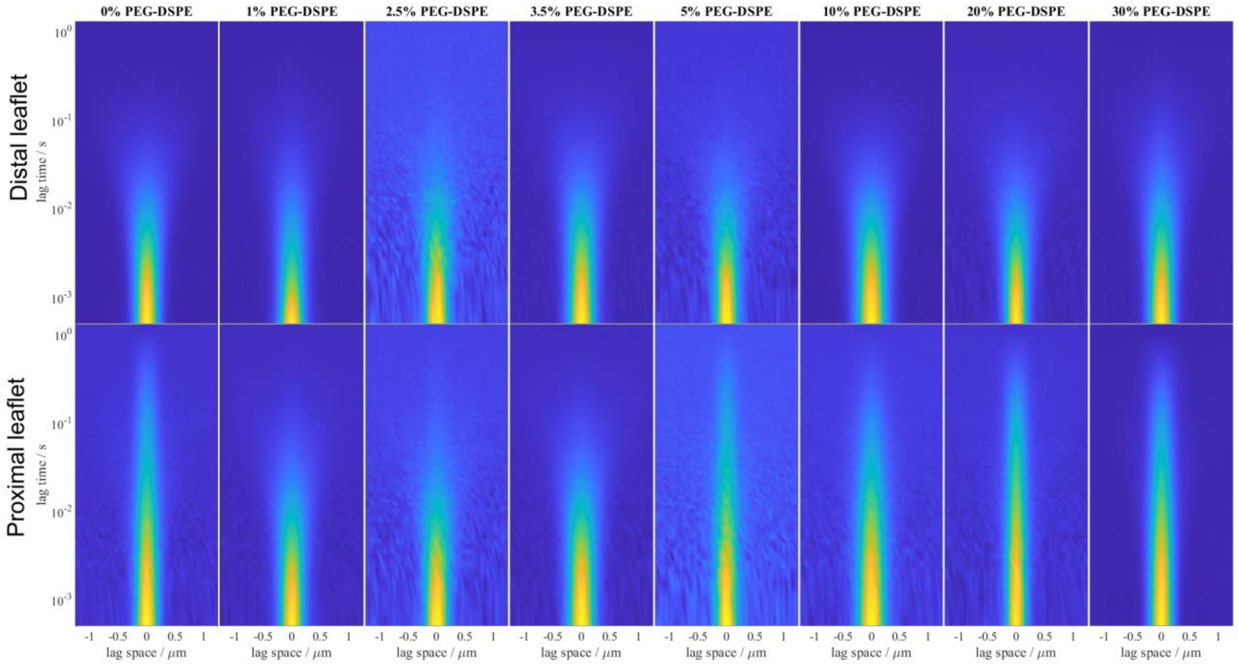

Figure S7. The calculated autocorrelation function for the distal leaflets and proximal leaflet for the PEG-SLB with varying concentrations.

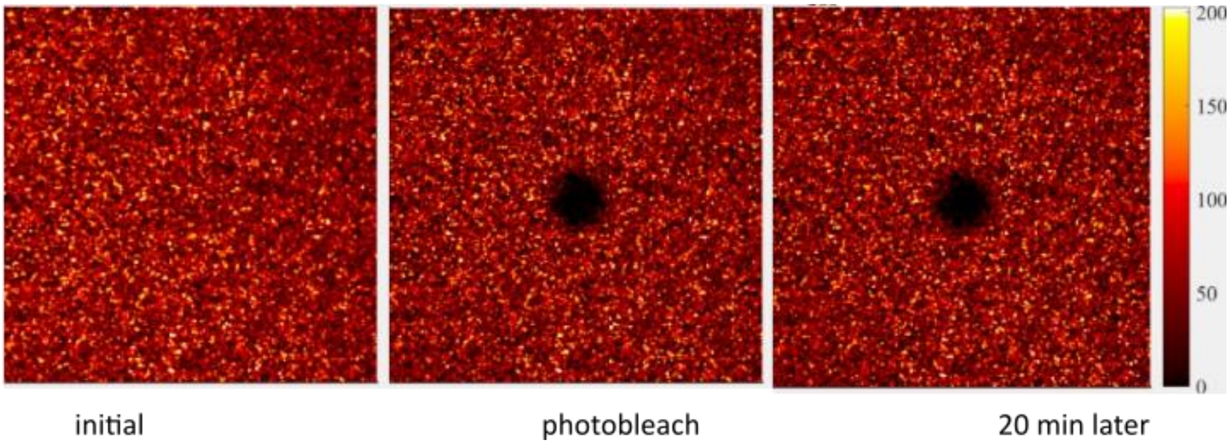

Figure S8. This figure demonstrates that the sample containing 40 wt% PEG2000-PE in DOPC exhibits no mobility. After photobleaching via illumination with strong laser intensity, the fluorescence does not recovery even after 20 min.

Table S1 Measured heigh, thickness, diffusion coefficient of distal and proximal leaflets for all samples.

Sample	Height (nm)	Thickness (nm)	D_{distal} (μm/s)	$D_{proximal}$(μm/s)
Substrate-SLB	1.3 ± 0.2	5.0 ± 0.08	1.95 ± 0.12	0.23 ± 0.06
1 wt% PEG-SLB	2.6 ± 0.1	4.6 ± 0.3	1.71 ± 0.16	1.23 ± 0.07
2.5 wt% PEG-SLB	3.2 ± 0.1	4.5 ± 0.3	1.86 ± 0.07	1.37 ± 0.16
3.5 wt% PEG-SLB	2.9 ± 0.2	4.4 ± 0.4	1.85 ± 0.1	1.38 ± 0.07
5 wt% PEG-SLB	3.0 ± 0.1	4.8 ± 0.2	2.03 ± 0.13	0.26 ± 0.15
10 wt% PEG-SLB	2.8 ± 0.2	5.4 ± 0.2	2.09 ± 0.12	0.17 ± 0.05
20 wt% PEG-SLB	3.0 ± 0.1	6.3 ± 0.4	2.12 ± 0.31	0.13 ± 0.09
30 wt% PEG-SLB	2.8 ± 0.2	7.8 ± 1.2	1.62 ± 0.41	0.19± 0.52
t-PEG-SLB	8.5 ± 0.1	5.1 ± 0.3	2.28 ± 0.09	1.82 ± 0.15
PEG-c-SLB	6.2 ± 0.3	5.0 ± 0.2	2.33 ± 0.11	1.98 ± 0.15

Reference

[1] F. Roder, S. Waichman, D. Paterok, R. Schubert, C. Richter, B. Liedberg, J. Piehler, *Anal. Chem.* **2011**, *83*, 6792–6799.

[2] C. Daniel, K. E. Sohn, T. E. Mates, E. J. Kramer, J. O. Rädler, E. Sackmann, B. Nickel, L. Andruzzi, *Biointerphases* **2007**, *2*, 109–118.

[3] T. Chen, A. Ghosh, J. Enderlein, *Nano Lett.* **2023**, *23*, 2421–2426.

[4] A. Ghosh, A. Sharma, A. I. Chizhik, S. Isbaner, D. Ruhlandt, R. Tsukanov, I. Gregor, N. Karedla, J. Enderlein, *Nat. Photonics* **2019**, *13*, 860–865.

[5] A. Ghosh, A. I. Chizhik, N. Karedla, J. Enderlein, *Nat Protoc* **2021**, *16*, 3695–3715.

[6] A. Ghosh, S. Isbaner, M. Veiga-Gutiérrez, I. Gregor, J. Enderlein, N. Karedla, *J. Phys. Chem. Lett.* **2017**, *8*, 6022–6028.

[7] T. Chen, N. Karedla, J. Enderlein, *Nat Commun* **2024**, *15*, 1789.

[8] J. Ries, S. Chiantia, P. Schwille, *Biophys J* **2009**, *96*, 1999–2008.
